# A Randomized, Double-Blind, Placebo-Controlled Study on Probiotic Treatment for Halitosis: Novel Insights into Glucose and Phosphorus Metabolism

**DOI:** 10.1007/s12602-025-10603-5

**Published:** 2025-06-13

**Authors:** Ji Hye Choi, Sehyeon Song, Min Ji Jang, Md Ariful Haque, Hye Eun Lee, Da Hui Kim, Yeo Ju Kim, Ja Won Cho, Jin Seok Moon, Keon Heo, Myeong Soo Park, Seockmo Ku

**Affiliations:** 1 Research Center, BIFIDO Co., Ltd, Hongcheon, 25117 South Korea; 2 Department of Food Science and Technology, Texas A&M University, College Station, TX 77843 USA; 3 Department of Dental Hygiene, Andong Science College, Andong, South Korea; 4 Department of Oral Health, Graduate School of Health and Welfare, Dankook University, Cheonan, South Korea; 5 Department of Preventive Dentistry, College of Dentistry, Dankook University, Cheonan, South Korea

**Keywords:** Halitosis, Oral probiotics, Paraprobiotics, *Lactobacillus gasseri*, *Lactobacillus paracasei*, Randomized controlled trial, Glucose, Phosphorus metabolism

## Abstract

Halitosis, or bad breath, is associated with oral microbial imbalances and the production of volatile sulfur compounds (VSCs). While existing treatments target pathogenic bacteria or oral health indicators, they may not address the underlying systemic complexity. This study explored the efficacy of Complex OK oral probiotics containing *Lactobacillus gasseri* HHuMIN D and *L. paracasei* OK in mitigating halitosis by evaluating VSC levels and metabolic markers. A 12-week, randomized, double-blind, placebo-controlled clinical trial was conducted involving 80 participants, 70 of whom completed the study in South Korea (KCT0009894). The participants were selected based on the presence of 2 of 3 pathogenic oral bacteria (*F. nucleatum*, *P. gingivalis*, and *P. intermedia*) and baseline VSCs > 2.0 ng/10 mL. Exclusion criteria included systemic diseases, recent antibiotic/probiotic use, and severe dental conditions. Oral health, VSCs, and metabolic markers were assessed using paired *t*-tests, ANCOVA, and Wilcoxon rank-sum tests. Despite unchanged oral health indicators and levels of harmful bacteria, probiotic supplementation showed efficacy in maintaining microbial balance. Significant reductions in H₂S and total VSCs were observed in the experimental group compared to the placebo (*P* < 0.05). No significant changes were observed in oral health indices or levels of harmful oral bacteria, but the experimental group showed a significant decrease in blood glucose (*P* = 0.009) and an increase in phosphorus levels (*P* < 0.05). This study provides the first published evidence linking systemic metabolic regulation to halitosis reduction, suggesting that probiotics mitigate bad breath through glucose and phosphorus metabolism rather than by direct bacterial inhibition. Further research is needed to confirm these findings and to explore the underlying mechanisms.

## Introduction

Halitosis (bad breath) is a common issue that affects more than half the global population [1, 2] with significant adverse impacts on individuals’ social relationships and mental health. The primary cause of halitosis is the production of volatile sulfur compounds (VSCs) in the oral cavity by resident anaerobic bacteria, particularly the gram-negative anaerobic species *Porphyromonas gingivalis* and *Treponema denticola* [3].

Two VSCs, hydrogen sulfide (H₂S) and methyl mercaptan (CH₃SH), are the key contributors to intraoral halitosis. These compounds are produced through bacterial metabolism of the sulfur-containing amino acids L-cysteine and L-methionine [2] with the generation of H₂S arising largely from bacterial enzyme reactions that degrade L-cysteine [2]. VSC-producing bacteria can be isolated from subgingival plaque in individuals with gingivitis or periodontitis and from saliva and the dorsum of the tongue in healthy individuals [4].

In the past, antibiotics have been widely used to address halitosis; however, due to their side effects and disruption of the normal oral microbiome, their use has become less common. Instead, research has increasingly focused on probiotic treatments that utilize beneficial microorganisms to inhibit the harmful oral cavity bacteria that are responsible for halitosis [5]. Although research on the use of probiotics to suppress halitosis is still in its early stages, some positive outcomes have been observed. For example, *Lactobacillus gasseri* HHuMIN D showed an 88.8% inhibitory effect on halitosis-causing bacteria [5]. Similarly, studies on *Lactobacillus plantarum* CCFM1214 and *Lactobacillus salivarius* CCFM1215 demonstrated significant reductions in VSC levels [6]. Additionally, *Streptococcus salivarius* K12 was found to reduce VSC levels when consumed as probiotic lozenges following a chlorhexidine pretreatment [7]. Moreover, the *S. salivarius* K12 and M18 strains were both effective in reducing the production of H_₂_S and CH₃SH [8]. However, not all probiotics are equally effective. For example, a study on *Lactobacillus casei* Shirota milk did not show significant changes in breath VSC concentrations or organoleptic scores [1]. The differences in outcomes may reflect variations in probiotic strains and research methodologies. For instance, [9] found that *S. salivarius* K12 did not significantly reduce halitosis if the tongue coating was not first removed by physical or chemical treatments, emphasizing the importance of tongue cleaning [9]. Proper oral hygiene using lingual cleaning devices and supplemented with probiotics can help prevent and treat oral malodor by restoring the normal oral microbiota, reducing dysbiosis, and suppressing gram-negative bacteria, thereby improving clinical indices [10]. Furthermore, the combined use of paraprobiotic-based toothpaste and mousse has demonstrated improved plaque control and a reduction in bleeding on probing (BoP), particularly in pregnant women [11].

In South Korea, research has actively examined halitosis, particularly during the COVID-19 pandemic, when prolonged mask wearing exacerbated halitosis by promoting anaerobic bacterial growth [12]. Research on adolescents found that 23.6% of the participants reported subjective halitosis, which was subsequently linked to factors such as stress, economic status, and dietary habits [13]. Another study revealed that women, rather than men, were more likely to experience halitosis but with notable age group differences [14]. Furthermore, studies on students have shown that halitosis can negatively affect mental health, leading to anxiety and stress [15, 16]. These findings have focused new attention on probiotics as treatments for halitosis.

In the USA, studies on probiotic products by [17] showed that chewing gum that contained probiotics had a positive impact on reducing halitosis by targeting the VSCs generated by VSC-producing bacteria as well as other malodorous compounds [17]. However, additional research is needed to standardize study designs and confirm the efficacy of probiotics as halitosis treatments [18–21]. The objective of the present study was to conduct a randomized, double-blind, placebo-controlled trial to investigate the efficacy of the *Lactobacillus gasseri* HHuMIN D and *Lactobacillus paracasei* OK combination (Complex OK) in reducing halitosis. Recent studies (e.g., [22]) have suggested a physiological interplay between blood glucose and phosphorus levels, with an inverse relationship often observed. In disease conditions such as diabetes, elevations in blood glucose can promote systemic inflammation and impair wound healing, both of which are associated with an increased risk of halitosis [23]. Consequently, glycemic control is considered important in managing bad breath in diabetic patients. While no direct evidence has yet linked blood phosphorus levels to halitosis, the metabolic connection between glucose and phosphorus suggests a potential indirect role. In this context, probiotic-induced changes in glucose and phosphorus metabolism may offer a novel systemic approach to reducing halitosis.

This study is the first to explore the potential of probiotics for reducing halitosis by targeting systemic metabolic pathways, specifically through the regulation of glucose and phosphorus metabolism. The findings identify a novel mechanism that extends beyond the traditional viewpoint that focuses on the oral microbial balance.

## Materials and Methods

### Ethics and Study Design

This study adhered to the Consolidated Standards of Reporting Trials (CONSORT) guidelines [24] and was conducted according to the World Medical Association Declaration of Helsinki (Version 2013). All subjects gave their written informed consent before participating in the study. The study protocol was approved by the Institutional Review Board of Dankook University Dental Hospital (DKUDH IRB 2022–6-003) and was registered at the Clinical Research Information Service (CRIS) as No. KCT0009894.

This study was designed as a randomized, double-blind, placebo-controlled, parallel clinical trial. Participants who voluntarily signed the informed consent form underwent demographic surveys, lifestyle assessments, medical and medication history evaluations, physical examinations, vital sign checks (blood pressure, pulse), anthropometric measurements (height, weight), clinical pathology tests, pregnancy tests (for women of childbearing age), panoramic X-rays, VSC measurements, and oral microbiome analyses. Participants deemed eligible based on the study inclusion and exclusion criteria were randomly assigned to the experimental or placebo groups. Participants were instructed to consume either the test product or a placebo, based on a 1:1 allocation ratio, for 12 weeks.

The study was planned for a 12-month duration starting from the approval date, with the first screening conducted on December 19, 2022, and the final participant visit on November 28, 2023.

### Participants

Participants were selected based on specific inclusion and exclusion criteria. The inclusion criteria required that the participants be adults aged 19–70 years, possess at least two of three harmful bacteria (*Fusobacterium nucleatum*, *Porphyromonas gingivalis*, or *Prevotella intermedia*) as identified through oral microbiome analysis, have VSC levels (hydrogen sulfide, methyl mercaptan, and dimethyl sulfide) ≥ 2.0 ng/10 mL in the VSC test, and provide written informed consent for participation. Exclusion criteria included individuals undergoing treatment for serious diseases (e.g., cardiovascular, immune, or respiratory), those with a history of systemic diseases known to cause bad breath, individuals with severe dental diseases, smokers, and individuals with other specific health conditions that could interfere with the study outcomes. A total of 80 participants were recruited, with the aim of obtaining data from at least 60 participants to ensure robust statistical analysis.

### Sample Size Calculation

The primary objective of this study was to evaluate whether VSC levels in the experimental group significantly decreased compared to the placebo group after 12 weeks. Based on a prior study [25], the required sample size was calculated using the mean difference and standard deviation of the VSC levels. A minimum of 30 participants per group was necessary to account for potential dropout rates, leading to a total recruitment of 80 participants.

### Intervention and Dosage

Participants in the experimental group consumed one tablet containing *L. gasseri* HHuMIN D and *L. paracasei* OK (1.0 × 10⁹ CFU/day) daily before bedtime, while the placebo group consumed an identical placebo tablet containing no probiotics. The intervention period lasted for 12 weeks.

### Outcome Measures and Methods

The primary outcome measure was the concentration of volatile sulfur compounds (VSCs), including hydrogen sulfide (H_2_S), methyl mercaptan (CH_3_SH), and dimethyl sulfide ((CH_3_)_2_S). VSC concentrations were measured using Oral Chroma equipment. Measurements were taken once per visit, starting after the subjects refrained from speaking for 5 min. Measurements were conducted in a fasting state (at least 8 h) in the morning, without any tooth brushing or fluid intake. The 1-mL syringe used for intraoral gas collection was inserted deep into the subject’s mouth, ensuring that the tip did not come into contact with the tongue or saliva. The subjects kept their mouths closed during the collection of the intraoral gas sample.

The secondary outcome measures included the results from the Tongue Plaque Index (TPI), Gingival Index (GI), Plaque Index (PI), and Patient Hygiene Performance (PHP) Index. Oral microbiome analysis and organoleptic assessments of breath odor were also performed to compare improvements between the experimental and placebo groups.

### Primer and Probe Design

Primers and probes specific to *P. gingivalis*, *F. nucleatum*, *S. mutans*, *P. intermedia*, *L. gasseri* HHuMN D, and *L. paracasei* OK were designed based on target genes selected through Basic Local Alignment Search Tool (BLAST) analysis to ensure specificity. Each probe was labeled with either 6-carboxyfluorescein (FAM) or Hexachlorofluorescein (HEX) as fluorescent reporters at the 5′ end and quenched with Black Hole Quencher 1 (BHQ1) at the 3′ end to minimize background fluorescence. Primers and probes were synthesized by Macrogen (Seoul, Korea), and their sequences are shown in Table 1.

**Table 1:** Oligonucleotides used as primers and probes for digital PCR

Bacteria name	Oligonucleotide	Sequence (5′−3′)	Length (bp)	Reference
*Porphyromonas gingivalis*	Forward Primer	ACACGGTGTATCGTGACGGC	21	[26]
	Reverse Primer	GCCGGCTGCGTACTTAACCT	20	
	Probe	HEX-CGACCTACCGCGATGCAGGA-BHQ1	23	
*Fusobacterium nucleatum*	Forward Primer	GGCTGTCGTCAGCTCGTGTC	20	[27]
	Reverse Primer	CTCATCGCAGGCAGTATCGC	20	
	Probe	FAM-AACGAGCGCAACCCCTTTCG-BHQ1	23	
*Streptococcus mutans*	Forward Primer	CAGCGCATTCAACACAAGCA	20	[28]
	Reverse Primer	TGTCCCATCGTTGCTGAACC	20	
	Probe	HEX-TGCGGTCGTTTTTGCTCATGG-BHQ1	23	
*Prevotella intermedia*	Forward Primer	TGGTTCGATAACGGCAGCAT	21	[29]
	Reverse Primer	ACGTAGCCAACAGCAGGAAA	20	
	Probe	FAM-TCCCCTCTCAAATTCTTCATAGTC-BHQ1	24	
*Lactobacillus gasseri* HHuMN D	Forward Primer	GCATTAATCCTAGCAGCACGC	21	This study
	Reverse Primer	TAGGCATTTGAGCACCTCCTT	21	
	Probe	FAM-ACCTCCTCTCAAGTTGTTGATTC-BHQ1	24	
*Lactobacillus paracasei* OK	Forward Primer	GCTGCAAAAAGCCATCGACA	20	This study
	Reverse Primer	CGATTGACCGTGATTTCGGC	20	
	Probe	HEX-GCATTAACGGCACTGAACCA-BHQ1	23	

### DNA Preparation

DNA was extracted from saliva samples collected using the SpeciMAX Stabilized Saliva Collection Kit (Thermo Scientific) and processed with the KingFisher Flex automated system (Thermo Fisher Scientific). The automated extraction process involved multiple washing and elution steps to ensure high-quality DNA for digital PCR (dPCR). A 400 µL saliva sample was added to 50 µL of MagMAX Viral/Pathogen Ultra Enzyme Mix (Applied Biosystems) in a KingFisher Sample Plate. Deep-well 96-well plates were prepared for washing and elution. The washing plate was washed with 1000 µL of MagMAX Viral/Pathogen Wash Buffer (Applied Biosystems) and the elution plate was washed with 1000 µL of 80% ethanol. After washing the elution plate with 500 µL of 80% ethanol, 100 µL of MagMAX Viral/Pathogen Elution Solution was added to each well. The protocol included an enzyme digestion step at 60 °C for 15 min with medium-speed mixing, followed by DNA binding using 20 µL of MagMAX DNA/RNA Binding Beads and 530 µL of MagMAX Viral/Pathogen Binding Solution per well. Washing steps were performed sequentially to remove contaminants, and the magnetic beads were air-dried for 2 min. DNA was eluted into 100 µL of MagMAX Viral/Pathogen Elution Solution at 75 °C. DNA concentration and purity were assessed using a spectrophotometer (Nano-400 A).

### Digital PCR (dPCR) Conditions and Total Bacterial Count Calculation

The target bacterial genes were quantified using the QIAcuity digital PCR system (QIAGEN, Hilden, Germany). Reaction mixtures (15 µL) were prepared by combining 6.5 µL of RNase-free water, 3 µL of 4 × QIAcuity Probe Master Mix (QIAGEN), 0.5 µL of a primer–probe mix (forward primer, reverse primer, and probe at 10 pmol each), and 5 µL of template DNA (final concentration of 20 ng/µL). The plates were thermally cycled under the following conditions: Initial denaturation was performed at 95 °C for 2 min, followed by 50 cycles of denaturation at 95 °C for 15 s, annealing/extension at 60 °C for 30 s, and a final extension at 75 °C for 2 min. Total bacterial counts were calculated by determining the average concentration (copies/µL) in duplicate wells for each target, and the result was multiplied by the template dilution factor to account for sample preparation. The total saliva weight was determined by subtracting the average kit weight from the total weight of the collection kit and converting it to mg of saliva by multiplying by 1000. The concentration of each bacterial species was then converted to total bacterial counts per g of saliva using standard curve equations specific to each target, providing the total bacterial counts for all target species.

### Statistical Analysis

Data were analyzed using SAS® (Version 9.4), with a significance level of 0.05 for two-sided tests. The main outcome measures were analyzed using paired *t*-tests and ANCOVA to compare the experimental and placebo groups, adjusting for demographic differences when necessary.

### Safety Evaluation

Safety was assessed by monitoring the incidence of adverse events, vital signs (blood pressure, pulse), body weight, and clinical pathology test results throughout the study period.

## Results

### Participant Characteristics

**Fig. 1:**
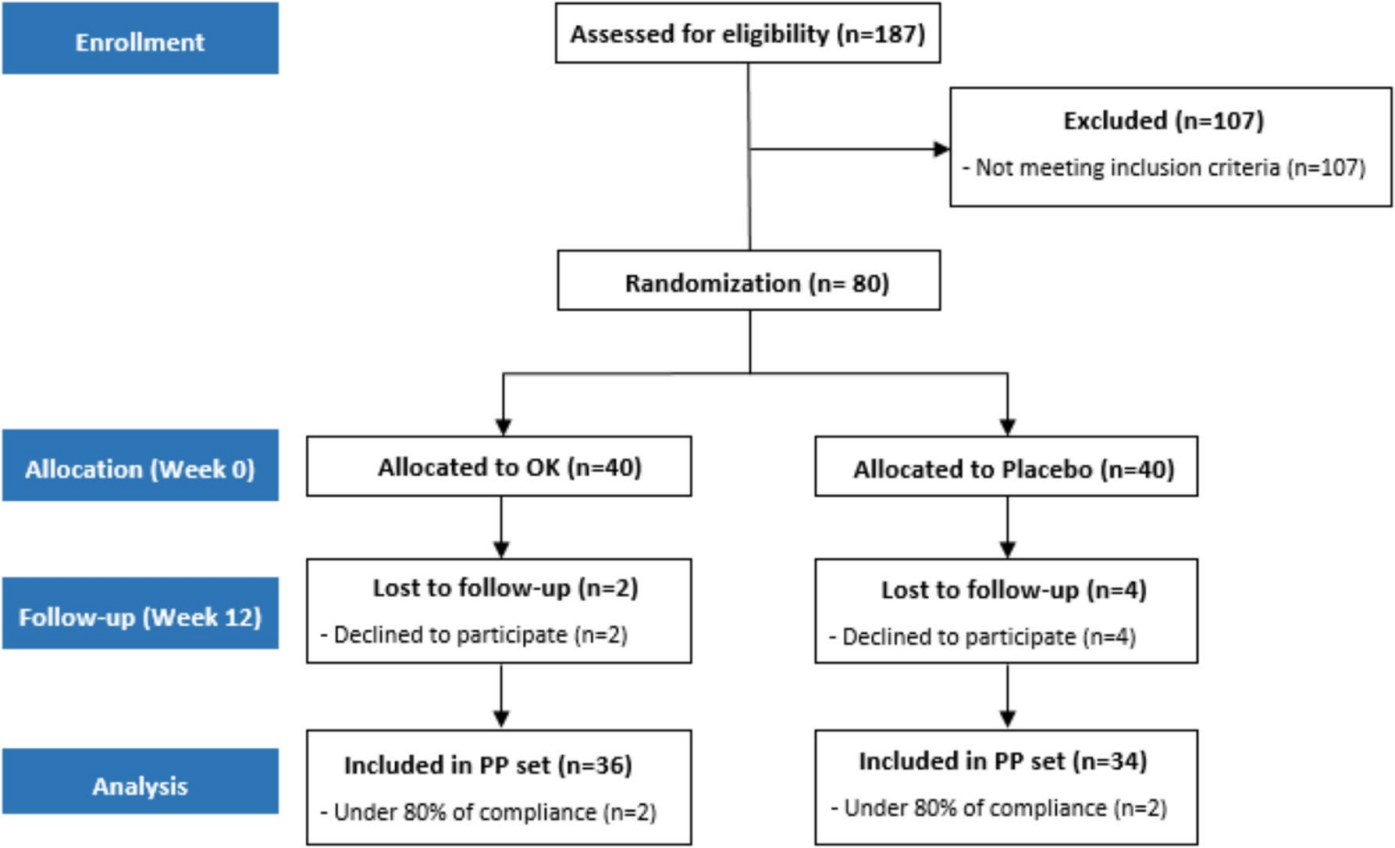
Consort diagram for the flow of subjects throughout the study

The clinical trial commenced on December 19, 2022, with the enrollment of the first subject, and concluded on November 28, 2023, with the enrollment of the last subject. A total of 187 applicants underwent a screening process to identify those who were deemed suitable candidates. Of the 187 individuals, 107 did not meet the established inclusion criteria and were excluded. Ultimately, 80 participants were randomly assigned (40 to the experimental group and 40 to the control group). Four participants (two from each group) withdrew from the study for personal reasons, resulting in a total of four dropouts. Six additional participants (two from the experimental group and four from the control group) were excluded from the analysis due to an average adherence rate below 80%. Ultimately, 70 participants (36 in the experimental group and 34 in the control group) were included in the final per-protocol analysis (Fig. 1, Table 2).

**Table 2:** Baseline characteristics of the study subjects

		OK (*n* = 36)	Placebo (*n* = 34)	*P*-value
Sex *n* (%)	Male	19 (52.78)	17 (50.00)	0.8162 (C)
	Female	17 (47.22)	17 (50.00)	
Age (years)		30.50 ± 11.54	30.38 ± 10.83	0.9110 (W)
Height (cm)		170.16 ± 10.63	168.99 ± 8.24	0.6091 (T)
Weight (kg)		69.63 ± 13.80	67.20 ± 12.63	0.4467 (T)
Drinker (yes/no)		29/7	24/10	0.3311 (C)
Self-perceived halitosis (yes/no)		22/14	19/15	0.6571 (C)

Comparison between groups; p-value for chi-square test (C), Two sample t-test (T) or Wilcoxon rank sum test (W)

### Oral Health Assessment

**Fig. 2:**
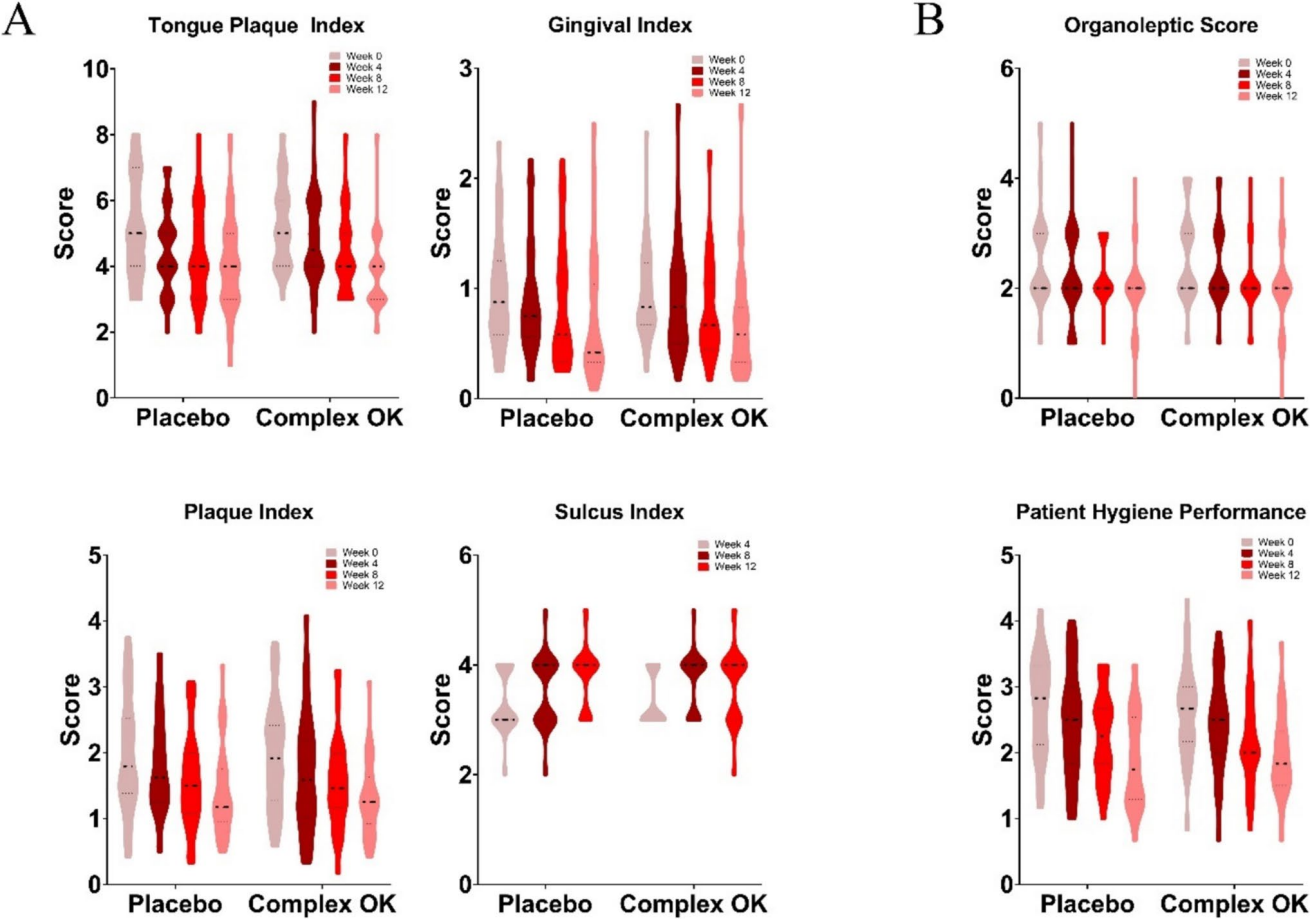
**A** Comparison of oral hygiene indicators between the placebo group (control) and the Complex OK group (test) over the course of the clinical trial. Metrics included the Tongue Plaque Index (TPI), Gingival Index (GI), Plaque Index (PI), and Sulcus Index (SI). **B** Clinical outcomes assessing halitosis (organoleptic score) and patient hygiene performance across different time points

Scores for oral health parameters, including the Tongue Plaque Index (TPI), Gingival Index (GI), Plaque Index (PI), Sulcus Index (SI), Organoleptic Score (OS), and Patient Hygiene Performance (PHP), were assessed every 4 weeks from baseline to week 12 for both the placebo and Complex OK groups (Fig. 2A, B). No statistically significant differences were observed between the groups across all parameters. These findings indicate that while the oral Complex OK probiotic acted under controlled conditions to influence tongue coating, gums, plaque, and other indices, it did not yield statistically significant differences.

### Oral Microorganisms

**Fig. 3:**
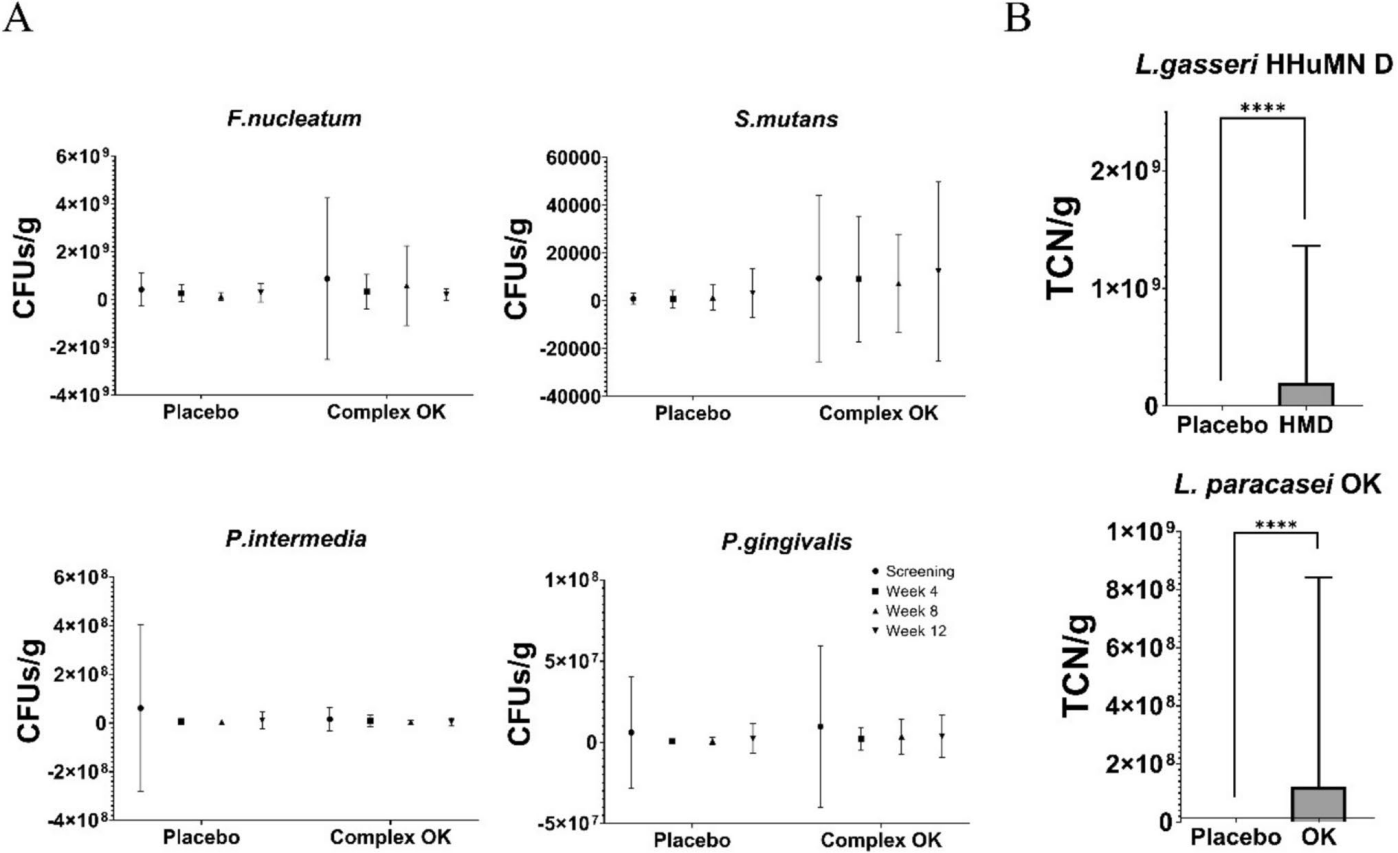
**A** Survival outcomes of *Fusobacterium nucleatum*, *Streptococcus mutans*, *Prevotella intermedia*, and *Porphyromonas gingivalis* across different dental visits in the placebo and Complex OK groups. **B** Confirmation of the presence of Complex OK strains, including *Lactobacillus gasseri* HHuMN D and *L. paracasei* OK, with significant differences observed (*P* < 0.0001) between the placebo and test groups

Due to the inherent variability of clinical data, large error bars were observed, particularly in the Complex OK group. This reflects individual patient differences in microbial responses to probiotic interventions, as is commonly encountered in human studies. The levels of four representative oral pathogenic microorganisms (*F. nucleatum*, *S. mutans*, *P. intermedia*, and *P. gingivalis*) were measured every 4 weeks (Fig. 3A). Although no statistically significant differences were observed between the placebo and Complex OK groups, a slight downward trend in *F. nucleatum* and *P. gingivalis* was noted in the Complex OK group over time, whereas these pathogen populations remained stable in the placebo group. In contrast, the *S. mutans* and *P. intermedia* levels remained relatively unchanged across both groups. These findings suggest that the probiotic intervention did not significantly disrupt the oral microbial environment. The Complex OK organisms were detected in the oral cavity and remained present in the Complex OK group through week 12 (Fig. 3B), indicating successful colonization without major shifts in microbial composition.

### Volatile Sulfur Compounds (VSCs)

**Fig. 4:**
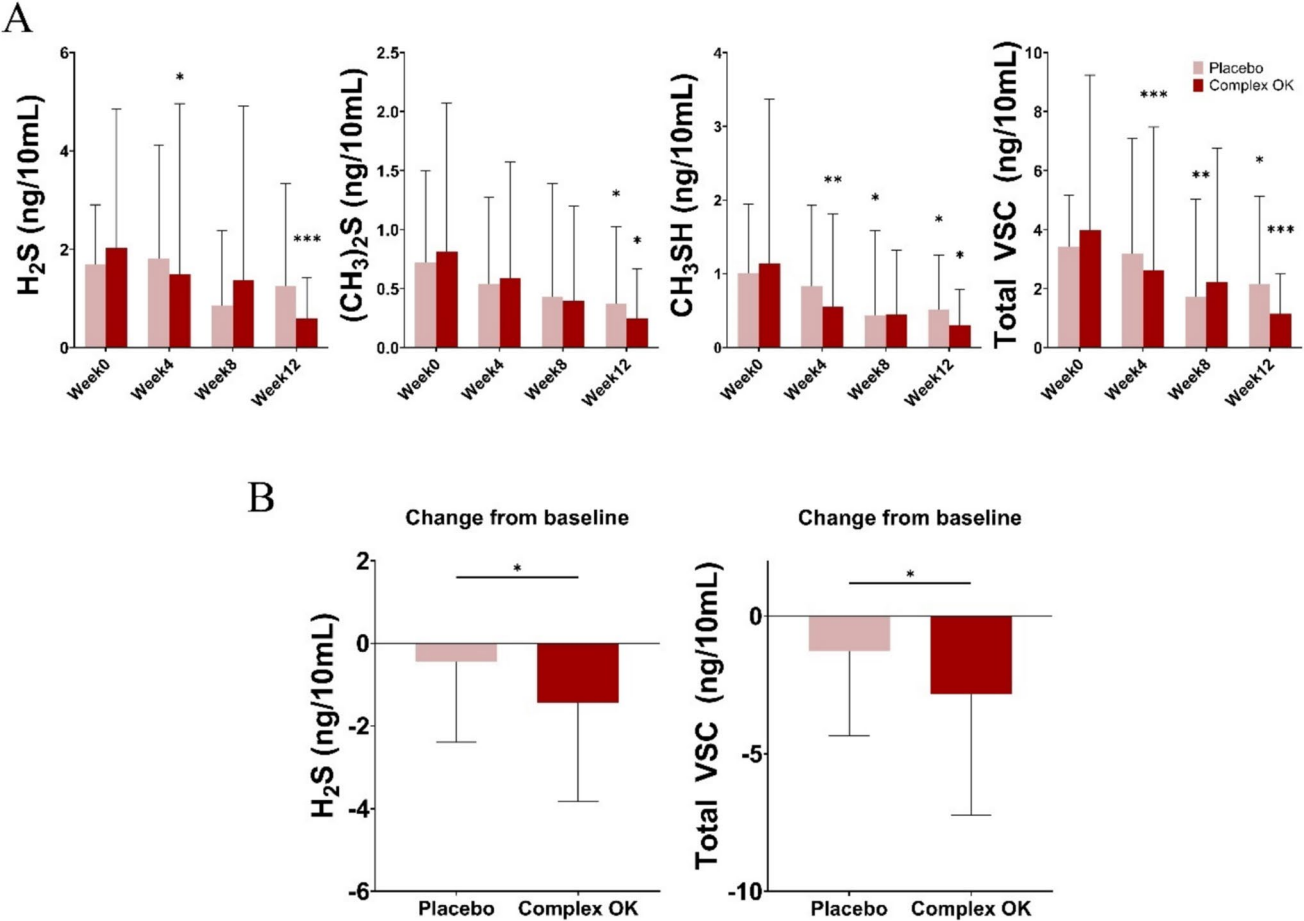
**A** Levels of volatile sulfur compounds (H₂S, (CH₃)₂S, CH₃SH, and total VSC) at baseline, week 4, week 8, and week 12 for the placebo and Complex OK groups. Asterisks indicate statistically significant differences compared to baseline (week 0) within the same group (paired *t*-test; *P < 0.05, **P < 0.01, ***P < 0.001). **B** Changes in H₂S and total VSC levels from baseline, with significant reductions observed in the Complex OK group compared to the placebo group (*P < 0.05, **P < 0.01, ***P < 0.001, ANCOVA-adjusted for baseline values)

This analysis revealed significant reductions in VSCs in both the Complex OK and placebo groups, with greater reductions observed in the experimental group (Fig. 4A, B). For H_2_S, significant decreases were observed in the experimental group at week 4 (*P* < 0.05) and week 12 (*P* < 0.001), while total VSC levels also showed significant declines at both week 4 (*P* < 0.001) and week 12 (*P* < 0.001). The primary endpoint demonstrated a mean reduction in VSCs of − 2.83 ± 4.40 ng/10 mL in the experimental group (*P* = 0.0005), compared to − 1.28 ± 3.07 ng/10 mL in the placebo group (*P* = 0.0205), and the difference was statistically significant (*P* = 0.0355). Among the subcomponents, H_2_S exhibited the largest reduction in the experimental group (− 1.43 ± 2.39 ng/10 mL, *P* = 0.0010), with a significant difference compared to the placebo group (*P* = 0.0364). As illustrated in Fig. 4B, when evaluated as a change from baseline, the experimental group consistently demonstrated greater reductions in both H_2_S and total VSC levels compared to the placebo group. These results suggest that while improved oral hygiene contributed to VSC reductions in both groups, Complex OK supplementation provided an additional benefit by reducing H_2_S levels in particular (Table 3).

**Table 3:** Results for the endpoints at 12 weeks from baseline according to group

Indicator		Treatment	Baseline	12 weeks	Mean change	*P*-value [1]	Significance [1]	OK vs. Placebo *P*-value			
								*P*-value [2]	Significance [2]	*P*-value [3]	Significance [3]
VSC (ng/10 mL)	H_2_S	OK	2.02 ± 2.84	0.59 ± 0.83	− 1.43 ± 2.39	0.001	***	0.1982 (W)	-	0.0364	*
		Placebo	1.69 ± 1.21	1.26 ± 2.08	− 0.44 ± 1.95	0.1987	-				
	CH_3_SH	OK	1.14 ± 2.23	0.30 ± 0.49	− 0.83 ± 1.89	0.0122	*	0.7779 (W)	-	0.1137	-
		Placebo	1.01 ± 0.94	0.51 ± 0.74	− 0.50 ± 1.26	0.0281	*				
	(CH_3_)_2_S	OK	0.81 ± 1.25	0.25 ± 0.42	− 0.57 ± 1.35	0.0162	*	0.6340 (W)	-	0.3216	-
		Placebo	0.72 ± 0.78	0.37 ± 0.65	− 0.35 ± 0.89	0.0298	*				
	Total	OK	3.97 ± 5.27	1.14 ± 1.35	− 2.83 ± 4.40	0.0005	***	0.1710 (W)	-	0.0355	*
		Placebo	3.42 ± 1.75	2.14 ± 3.00	− 1.28 ± 3.07	0.0205	*				
TPI		OK	5.31 ± 1.28	3.78 ± 1.12	− 1.53 ± 1.28	< 0.0001	***	0.2558 (W)	-	0.3332	-
		Placebo	5.18 ± 1.57	4.00 ± 1.37	− 1.18 ± 1.64	0.0002	**				
GI		OK	0.94 ± 0.41	0.68 ± 0.49	− 0.27 ± 0.35	< 0.0001	***	0.8846 (T)	-	0.8594	-
		Placebo	0.96 ± 0.50	0.71 ± 0.57	− 0.25 ± 0.39	0.0005	***				
PI		OK	1.96 ± 0.81	1.29 ± 0.56	− 0.67 ± 0.56	< 0.0001	***	0.6122 (T)	-	0.4651	-
		Placebo	1.97 ± 0.87	1.39 ± 0.67	− 0.58 ± 0.86	0.0004	***				
Organoleptic test		OK	2.58 ± 0.84	1.97 ± 0.74	− 0.61 ± 0.99	0.0008	***	0.9802 (W)	-	0.7632	-
		Placebo	2.53 ± 0.90	1.91 ± 0.71	− 0.62 ± 1.04	0.0016	**				

[1] Comparison within groups; *P*-value for the paired t-test

[2] Comparison between groups; *P*-value for the two sample t-test(T) or Wilcoxon rank sum test(W)

[3] Comparison between groups; *P*-value for the ANCOVA-adjusted baseline

### Reductions in Blood Glucose and Phosphorus Levels

**Fig. 5:**
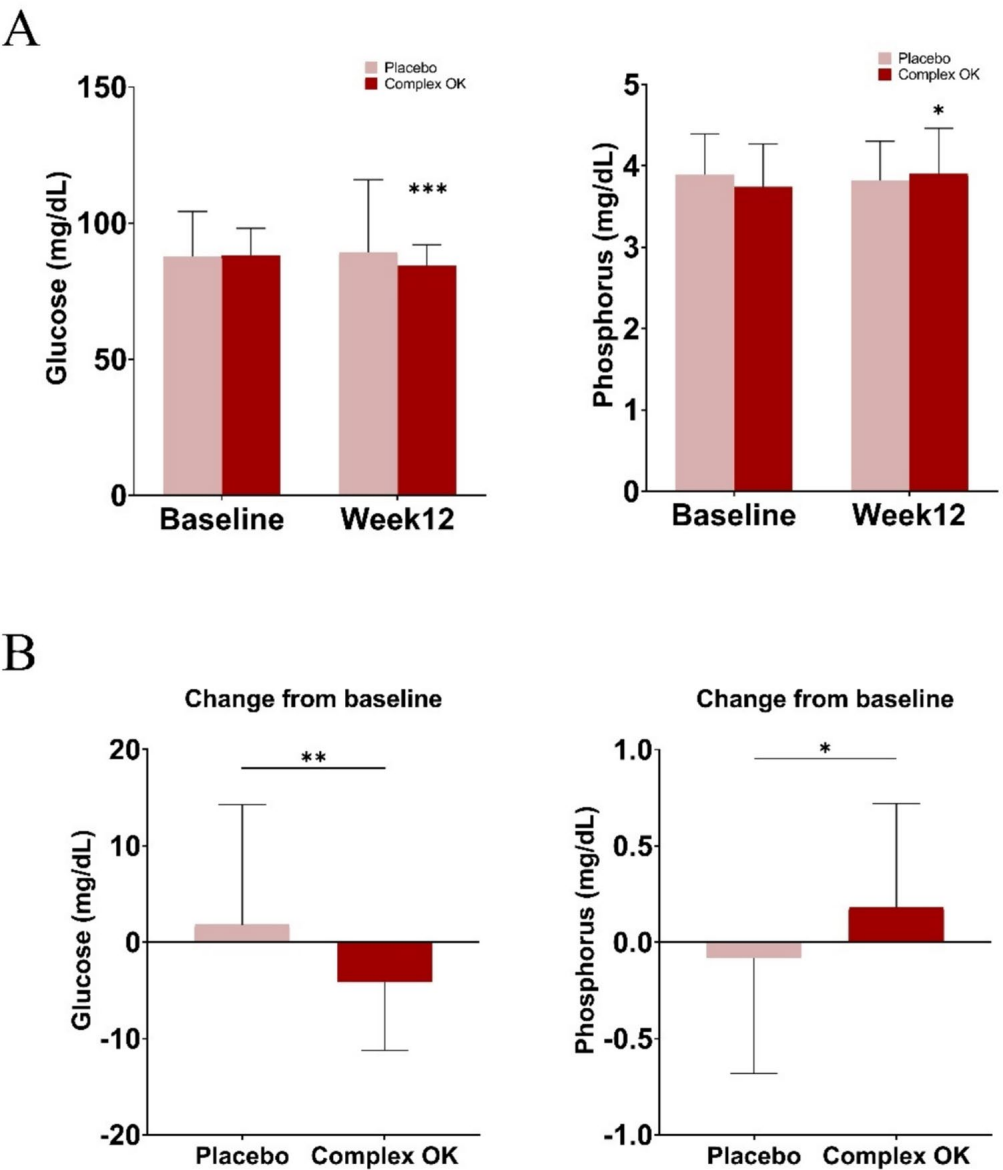
**A** Blood levels of glucose (mg/dL) and phosphorus (mg/dL) at baseline and week 12 for the placebo and Complex OK groups. Asterisks indicate statistically significant differences compared to baseline (week 0) within the same group as determined by paired *t*-tests. **B** Changes in glucose and phosphorus levels from baseline to week 12. Significant differences were observed in the Complex OK group compared to the placebo group using the Wilcoxon rank-sum test (*P < 0.05, **P < 0.01, ***P < 0.001)

Blood glucose and phosphorus levels were assessed at baseline and week 12 (Fig. 5A). At the final time point, the participants administered Complex OK demonstrated a significant reduction in blood glucose levels, with a decrease of 4.16 mg/dL compared to the baseline, whereas the placebo group exhibited an increase of 1.82 mg/dL (*P* = 0.0094). Phosphorus levels increased by 0.18 mg/dL in the Complex OK group (*P* < 0.05), while the placebo group showed a reduction of 0.08 mg/dL. The intergroup differences in phosphorus levels were also statistically significant (Fig. 5B).

### Safety

A safety evaluation was conducted for each subject who had been randomly assigned to the clinical trial and who had consumed the study product at least once (Safety Set), resulting in a total of 80 participants (40 in the experimental group and 40 in the control group). No adverse events were reported in either the experimental or control groups, and no statistically significant differences were observed between the groups. No serious adverse events or instances of dropout due to adverse events occurred. Clinical pathology assessments for safety, including hematological tests, blood chemistry tests, and urinalysis, were conducted during visits 1 and 5.

Among the blood chemistry tests, blood glucose levels exhibited a notable decline in the experimental group after 12 weeks (− 4.16 ± 7.07 mg/dL, *P* = 0.0009), whereas the control group demonstrated a nonsignificant increase (1.82 ± 12.45 mg/dL, *P* = 0.3744), resulting in a statistically significant discrepancy between the two groups (*P* = 0.0094 [W]). While some subjects in both groups exhibited values outside the normal range, no clinically meaningful cases were identified. This meant that no adverse events requiring medical intervention, discontinuation of the study, or deemed by investigators to be related to the test product were reported. Additionally, no significant differences were observed in the remaining blood chemistry tests. Hematological tests and urinalysis also showed no statistically significant differences between the two groups. Vital signs (pulse, blood pressure) and physical measurements (weight) remained within normal ranges in both groups after 12 weeks, with no statistically significant changes observed.

## Discussion

In this study, a significant reduction in hydrogen sulfide (H_2_S) and total volatile sulfur compounds (VSCs) was observed in the experimental group compared to the placebo group after 12 weeks of supplementation with Complex OK oral probiotics (*P* < 0.05 for H_2_S and *P* < 0.001 for total VSCs at week 12). While both groups exhibited reductions in VSC levels relative to the baseline, the significantly greater reduction in the experimental group confirms the efficacy of Complex OK in mitigating halitosis [32–34]. Although research on probiotics for halitosis indicates promising possibilities, clinical studies and scientific validation are still needed to establish probiotics as an effective and reliable bad breath treatment [35]. The aim of the present study was to determine whether the optimal microbial concentrations previously established to reduce volatile sulfur compound production in in vitro experiments (data not shown) would also do so in vivo in a 12-week clinical trial (the longest period to date) designed to ascertain whether statistically significant reductions would occur in the VSC concentrations as a primary outcome measure [36].

Measurements of diverse oral health indicators are critical in any study aimed at understanding halitosis, as its causes are diverse and can arise from multiple oral conditions. As noted in previous research, 90% of halitosis cases originate from oral health issues, including gum disease, periodontal disease, tongue disorders, and other oral health conditions [4, 37]. Therefore, this study employed clinical oral health indicators to identify the underlying causes of halitosis. Many previous studies have demonstrated improvements in the Tongue Plaque Index as an effective means of addressing halitosis-related disorders [9] or have observed associations between lower VSC levels and an improved Gingival Index [6]. Conversely, some studies have reported reduced subjective halitosis, but without significant improvements in oral health indicators, following the consumption of oral probiotics [15].

As shown in Fig. 2A, the consumption of Complex OK did not result in statistically significant differences in oral health indicators between the placebo and experimental groups. Changes in oral health indicators are often linked to shifts in the oral microbial composition, with harmful bacteria reduced and beneficial probiotics colonizing the oral cavity [6]. In the present study, as shown in Fig. 3B, the Complex OK strains successfully colonized the oral cavity. While a concomitant decreasing trend in harmful oral bacteria was observed, the changes were not statistically significant. These results suggest that Complex OK reduces VSCs through mechanisms other than direct improvements in oral health indicators or the eradication of harmful oral bacteria.

One possibility is that Complex OK functions by improving the oral environment and mitigating conditions favorable to the proliferation of harmful bacteria. This hypothesis is supported by the data presented in Fig. 4, which highlights reductions in VSCs independent of significant changes in the microbial load of harmful bacteria in the oral environment.

These findings emphasize the unique mode of action of Complex OK, distinguishing it from traditional approaches to halitosis management. The lack of significant changes in oral health indicators or pathogenic bacterial levels suggests that Complex OK reduces VSCs through mechanisms that extend beyond direct bacterial eradication and support its proposed “balancing effect” [38]. By promoting microbial homeostasis in the oral cavity, Complex OK mitigates conditions favorable to the proliferation of harmful bacteria while preserving the overall microbial ecosystem. However, the systemic metabolic changes observed in the participants in this study provide an alternative explanation for the reduction in VSCs.

Blood glucose levels decreased significantly by 4.16 mg/dL in the experimental group compared to a 1.82 mg/dL increase in the placebo group (*P* = 0.0094). Blood phosphorus levels increased by 0.18 mg/dL (*P* < 0.05) in the experimental group, while the placebo group showed a decrease of 0.08 mg/dL. Elevated blood glucose has been associated with increased oral malodor [39], suggesting that reduced blood glucose levels likely suppressed the metabolic activity of VSC-producing bacteria. Disruptions in glucose metabolism, such as in diabetes, are well known to lead to halitosis. This is because abnormal glucose metabolism often triggers inflammation, impairs wound healing, and increases the risk of periodontal diseases. These conditions create an environment that promotes the proliferation and metabolic activity of odor-causing bacteria, resulting in halitosis [31]. A strong association between halitosis and glucose metabolism is also evident from advancements in diagnostic technologies that use halitosis biomarkers to detect diabetes [40].

Phosphorus metabolism also plays a crucial role in cellular energy production and the stability of the oral mucosa [41]. The observed increase in blood phosphorus levels in the experimental group may have contributed to the stabilization of oral mucosal barriers, while also enhancing ATP production, to create an unfavorable environment that suppressed the metabolism of VSC-producing bacteria, thereby limiting the production of halitosis-causing compounds.

Our findings are consistent with previous research demonstrating the systemic effects of probiotics. For instance, *L. gasseri* and *L. paracasei* have been shown to improve glucose metabolism and phosphorus regulation in animal models [42, 43]. In addition, [44] reported that *L. plantarum* FMP-B719 normalized phosphorus levels in an ovariectomy-induced postmenopausal osteoporosis rat model, highlighting the role of phosphorus metabolism in systemic health [44]. The improvement in phosphorus metabolism observed in this study may have stabilized oral microbial interactions and contributed to the reduction in VSC production.

Previous research has shown that serum phosphorus levels are inversely related to glucose and insulin responses, and the co-ingestion of phosphorus with glucose has been demonstrated to improve postprandial glucose and insulin levels [22]. These findings are consistent with our observation of increased serum phosphorus and decreased blood glucose levels following Complex OK supplementation. This improved metabolic balance may have contributed to the reduction in VSC levels observed in this study.

Phosphorus plays a vital role in the development and maintenance of mineralized oral tissues, including enamel, dentin, cementum, and alveolar bone. Disruptions in phosphate homeostasis can negatively impact these tissues and contribute to the onset of periodontal disease [45]. Previous research has primarily focused on the role of phosphorus in tooth development and gingival regeneration. Elevated dietary phosphate intake has been linked to increased salivary concentrations of pro-inflammatory markers, such as interleukin-1β (IL-1β) and matrix metalloproteinase-9 (MMP-9), suggesting that excessive phosphorus consumption may directly contribute to halitosis [46]. However, in our study, while a significant increase in blood phosphorus levels was observed in the experimental group, this was not associated with worsened halitosis. Instead, our findings suggest that the elevated phosphorus levels may be linked to improved glucose metabolism, indicating a systemic regulatory effect rather than a direct pathogenic role in halitosis. The increase in phosphate availability was shown to enhance intracellular glucose phosphorylation, with insulin release being highly dependent on the circulating glucose concentration. This ultimately resulted in reduced blood glucose and insulin levels [47]. However, pre-ingestion of phosphorus 60 min prior to a glucose load did not have the same effect, likely because phosphorus is typically absorbed within an hour, and a decline in postprandial phosphorus levels during glucose loading is expected [22]. These findings suggest a physiological link between phosphate and glucose metabolism. While several studies have demonstrated a relationship between glucose and phosphorus metabolism, and between glucose levels and halitosis via volatile sulfur compound (VSC) production, direct evidence linking phosphorus metabolism to halitosis or VSC generation remains limited.

The findings of the present study suggest that Complex OK can regulate glucose metabolism and contribute to an overall reduction in halitosis. Therefore, Complex OK points to a novel strategy for halitosis management that extends beyond traditional approaches focused on oral health indicators or direct bacterial eradication to therapies designed to influence glucose and phosphorus metabolism. These findings highlight the importance of addressing systemic metabolic pathways, rather than the causative VSC-producing bacteria, in the treatment of halitosis.

The findings presented here also suggest that the systemic effects of Complex OK are likely to extend beyond the oral cavity. Approximately 10% of halitosis cases originate from the gastrointestinal tract, and the metabolic changes observed in this study suggest that Complex OK may reduce VSC production from nonoral sources by influencing the patient’s systemic metabolism and gut microbiota balance [23, 48]. This systemic mechanism aligns with the broader applications of probiotics as an alternative to antibiotics, as highlighted by [4], as probiotics can offer sustainable therapeutic benefits without disrupting the body’s microbial ecosystems [4, 49].

The oral cavity and the gut are closely interconnected, and alterations in the gut microbiota can influence the oral microbiome. Accordingly, microbiota-targeted therapies, such as fecal microbiota transplantation, synbiotics, prebiotics, and postbiotics, may also have beneficial effects on the oral microbial balance [30]. These interventions are known to promote microbial homeostasis, highlighting the need for further investigations into their potential applications in halitosis management. Several studies have highlighted the link between gut health and halitosis, particularly in cases in which gastrointestinal disorders are implicated [4, 50]. A recent retrospective single-center trial by [51] reported that probiotics use effectively reduced halitosis caused by small intestinal bacterial overgrowth. In that study, administration of *Bifidobacterium longum* to the study participants at a dose of 5 × 10⁷ CFU per gram for two months resulted in noticeable improvements in oral malodor [51].

The selected dosages and strain combinations are clearly important factors that determine probiotic efficacy. Before initiating clinical trials, in vivo studies can serve as valuable references for selecting an appropriate dose, ranging from safe to therapeutic levels. According to [36], most studies include a follow-up period of approximately 8 weeks. However, the administered doses vary greatly, typically ranging between 1 × 10⁸ and 2 × 10⁹ CFU, and the delivery forms can include tablets, powders, and chewing gums. The long-term efficacy of probiotic therapy for halitosis remains unclear, as most clinical trials to date have focused on short-term outcomes, typically ranging from 4 to 12 weeks [36]. Although meta-analyses and randomized controlled trials have demonstrated that several probiotics, such as *Lactobacillus salivarius*, *Lactobacillus reuteri*, *Streptococcus salivarius*, and *Weissella cibaria*, can significantly reduce VSC levels and improve organoleptic scores in the short term, evidence supporting sustained efficacy is limited. Therefore, future studies should incorporate extended follow-up periods and long-term monitoring to assess the durability of probiotic effects. Those types of studies would help determine whether continuous or repeated probiotic administration is required to maintain reductions in VSCs and oral malodor and would establish the optimal duration and dosing regimen for effective probiotic therapy.

Currently, no standardized clinical guidelines exist for the use of probiotics in managing halitosis due to the identification of numerous moderating factors, such as strain type, administration method (tablet, gargle, gum, or lozenge), duration of use, pretreatment conditions, and dosage. Therefore, identifying effective probiotic strains and developing standardized treatment protocols require more systematic research. Overall, the present evidence supporting the benefits of probiotics for oral health and halitosis suppression remains limited [35].

## Conclusions

This study demonstrated that Complex OK oral probiotics significantly reduced halitosis-related VSCs without altering the oral microbial diversity or health indices. These effects were associated with improvements in systemic glucose and phosphorus metabolism, suggesting the operation of a mechanism beyond direct bacterial inhibition. Further research is needed to validate these findings and to explore the long-term systemic effects of Complex OK in larger populations.

This study was previously published as a preprint on Research Square under the title, “A Randomized, Double-Blind, Placebo-Controlled Study on Probiotic Treatment for Halitosis: Novel Insights into Glucose and Phosphorus Metabolism.”

## Data Availability

No datasets were generated or analysed during the current study.
